# Targeting of interaction between BB0323-BB0238 informs new paradigms in Lyme disease therapeutics

**DOI:** 10.1371/journal.ppat.1013805

**Published:** 2026-01-02

**Authors:** Sandhya Bista, Kalvis Brangulis, Bibek Bhattachan, Shelby D. Foor, Michael H. Ronzetti, Sankalp Jain, Jenna Miller, Jothy Lachumy Subramanion, Chrysoula Kitsou, Vipin S. Rana, Ganesha Rai, Alexey V. Zakharov, Anton Simeonov, Bolormaa Baljinnyam, Utpal Pal

**Affiliations:** 1 Department of Veterinary Medicine, University of Maryland, College Park, Maryland, United States of America; 2 Latvian Biomedical Research and Study Centre, Riga, Latvia; 3 Department of Human Physiology and Biochemistry, Riga Stradins University, Riga, Latvia; 4 National Center for Advancing Translational Sciences, National Institutes of Health, Rockville, Maryland, United States of America; 5 Virginia-Maryland College of Veterinary Medicine, College Park, Maryland, United States of America; Centre National de la Recherche Scientifique, FRANCE

## Abstract

*Borrelia burgdorferi*, one of the most prevalent tick-borne pathogens, can cause a complex and multisystem illness called Lyme disease, where there has been an unmet need for novel therapeutic or preventive strategies. We previously identified an essential protein-protein interaction (PPI) event in *B. burgdorferi* involving two unique proteins, BB0323 and BB0238; herein, we show that this PPI is indispensable for long-term borrelial survival in mammals and explore its potential as a novel target for small molecule therapeutics. Using X-ray crystallography, we solved the structure of the BB0238-BB0323 complex and identified the hotspot residues that form the biomolecular PPI interface area of ~1000 square Ångstroms. We then performed quantitative high-throughput drug screens of 62,740 diverse small molecules utilizing an amplified luminescent proximity homogeneous assay linked immunosorbent assay (AlphaLISA). Following a comprehensive pipeline to confirm small molecule hits, we short-listed three distinct PPI inhibitors of BB0238-BB0323. One of these inhibitors, called lomibuvir (VX-222, VCH-222), displayed robust PPI inhibition inside *B. burgdorferi* cells and was shown to affect pathogen persistence in a tick-borne murine model of Lyme disease. Our study highlights targeted PPI disruption as a new therapeutic strategy against *B. burgdorferi* and may foster future antimicrobial discovery efforts to resolve clinical complications associated with Lyme disease.

## Author summary

*Borrelia burgdorferi*, a common tick-borne bacterial pathogen, causes a complex illness called Lyme disease, where a human vaccine is unavailable. Although early antibiotic treatment is effective, many patients develop relapsing symptoms unresponsive to further treatment. Therefore, a better understanding of the infection biology of Lyme disease, and the development of novel therapeutic strategies, is warranted. We leveraged a unique protein-protein interaction (PPI) in *B. burgdorferi* that is indispensable for borrelial survival in mammals as a novel small molecule therapeutic strategy. Using structural biology and high-throughput drug screening, we identified compounds that can inhibit the PPI and reduce infection. Our finding highlights targeted PPI disruption as a potential therapeutic strategy against *B. burgdorferi* infection.

## Introduction

The tick-borne pathogen *Borrelia burgdorferi*, which is the microbial agent of Lyme disease, causes one of the most common vector-borne infections in many parts of the world, primarily in Europe and North America [1]. The infection is now reported in more than 80 countries, and 476,000 new cases of Lyme disease are estimated yearly in the United States alone [2]. Due to the absences of human vaccines and optimal diagnostics for early infection, together with additional zoonotic factors, Lyme disease is on the rise [3]. A more comprehensive treatment strategy for Lyme disease remains an unmet need, as the currently available antimicrobial therapies are not always effective or complete. In fact, after standard-care antibiotic regimens, a fraction of patients experiences relapsing symptoms of variable intensity known as post-treatment Lyme disease syndrome [4]. Its etiology, pathogenic mechanisms, or treatment choices are virtually unknown, and additional antibiotic treatment fails to improve clinical complications [4–6]. The identification of novel therapeutic strategies for Lyme disease, including the discovery of new antimicrobials, therefore remains a highly warranted goal of Lyme disease research.

*B. burgdorferi* belongs to an evolutionarily distinct group of pathogens that is remarkably adapted to thrive in nature through a complicated tick-mammal enzootic infection cycle [7]. The bacterium features an unorthodox genome with many extrachromosomal genetic elements, in addition to unconventional outer membrane and cellular architecture and compositions; thus, it can be considered an atypical gram-negative bacterial species [8–10]. Much of the *B. burgdorferi* genome encodes gene-products that have unknown functions or structures yet are essential for infection [8,9]. Therefore, it is likely that these spirochetes evolved unique mechanisms to persist in multiple disparate environments, spanning the tissues of vertebrate hosts and arthropod vectors like *Ixodes scapularis*, and to survive long-term in mammals, including incidental hosts like humans, even after available antibiotic therapy.

Our recent studies identified two key *B. burgdorferi* virulence factors, annotated as BB0323 and BB0238, as multidomain and multifunctional proteins [11–16]. BB0323 is proteolytically cleaved into its mature form as N-terminal and C-terminal polypeptides [13], the former of which forms a protein complex with BB0238 [12,15]. Our previous structural analyses [17] showed that the overall fold of the N-term BB0323 belongs to the spectrin repeats protein family that is rare in bacteria. While BB0238 has similarities to transport and/or chaperone proteins [11], it is also involved in a protein-protein interaction (PPI) with BB0323, which was found to facilitate their posttranslational stability and is essential for microbial persistence in mammalian hosts and transmission between ticks and mammals [12,14,15].

Herein, we report that the BB0238-BB0323 PPI is critical for long-term spirochete persistence in the host and pathogen transmission to the tick vector. We used multidimensional approaches, including X-ray crystallography to solve the 3D structure of the BB0238-BB0323 complex, as well as quantitative high-throughput robotic screens of 62,740 compounds of diverse chemotypes to identify BB0238-BB0323 PPI inhibitors. In an effort to inform the future development of next-generation therapeutics that target essential PPI events, we also present proof-of-concept evidence that one of the shortlisted BB0238-BB0323 inhibitors, lomibuvir, impacts spirochete infectivity in murine hosts, thereby highlighting a new paradigm for the treatment of Lyme disease.

## Results

### BB0238-BB0323 interactions are indispensable for *Borrelia burgdorferi* long-term persistence in mammals

**Fig 1:**
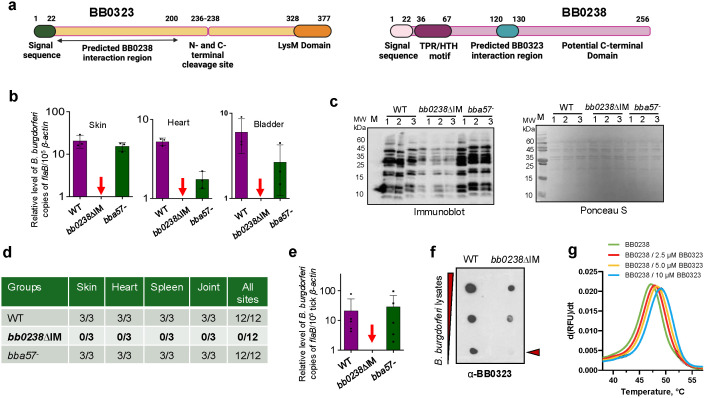
BB0323-BB0238 interaction is essential for long-term persistence of *B. burgdorferi* in mice. **(a)** Schematic representation [81] of the primary structure of full-length BB0323 (left panel) and BB0238 (right panel) proteins. The signal sequences, domains, and motifs, as well as the predicted PPI site, are shown. **(b-e)** Impaired BB0323-BB0238 interaction diminishes long-term spirochete persistence in mice. Mice were infected with equal numbers of wild type (WT) *B. burgdorferi*, PPI deficient (*bb0238∆IM*), or isogenic mutant control (*bba57-*) isolates, and pathogen levels were analyzed after nine weeks using various modalities, such as RT-qPCR analyses of murine tissues by measuring *B. burgdorferi flaB* transcript level normalized to mouse *β-actin* level **(b)**; immunoblot analyses using sera from infected mice where individual lanes were excised, processed, and subsequently reassembled to a composite image **(c, left)** and Ponceau S staining as control for total protein load **(c, right)**; and culture analysis for re-growth of viable spirochetes from indicated tissue samples **(d)**. Xenodiagnosis studies were performed to determine if ticks acquired spirochetes from infected mice, as assessed by RT-qPCR, where *flaB* levels were normalized to tick *β-actin* **(e)**; red arrows denote undetectable *bb0238∆IM* levels, and bars represent the mean ± SEM of four RT-qPCR analyses from two independent animal experiments. **(f)** Reduced presence of BB0323 in PPI-deficient mutants compared to wild type (WT), as shown by a simple dot-blot assay. Serial dilution of *B. burgdorferi* lysates were immunoblotted with anti-BB0323 antibody. **(g)** Enhanced stability of 20 µM BB0238 in the presence of increasing BB0323 levels. nanoDSF unfolding profile of BB0238 shown by the first derivative of fluorescence intensity in response to temperature. *p < 0.05.

Spirochetes of the *Borrelia burgdorferi* sensu lato complex produce conserved proteins that are essential for infection, including BB0323 and BB0238, which feature identifiable motifs (Fig 1a) yet show overall weak homology to cytoskeletal and transport/chaperone proteins. In addition to the known requirement of BB0238-BB0323 protein-protein interactions (PPIs) for early stages of spirochete infection in mammals [15], we sought to assess the biological significance of this PPI in supporting the long-term persistence of *B. burgdorferi* in mammals. We used a previously-generated *B. burgdorferi* mutant termed *bb0238∆IM* [15], where the 11 amino acid BB0323-BB0238 interaction motif in BB0238 is mutated to alanine residues, rendering it PPI deficient. C3H mice were inoculated subcutaneously with either the wild type (WT) *B. burgdorferi*, *bb0238∆IM*, or a control isogenic mutant *bba57-* [18], and after nine weeks of infection, the pathogen burdens were evaluated. Quantitative reverse-transcription PCR (RT-qPCR) showed that spirochetes were undetectable in the *bb0238∆IM-*infected mouse tissues, compared to wild-type and *bba57-* mutants which were readily observable (Fig 1b). The murine hosts developed robust humoral immune responses against wild type and *bba57-* mutants, as detected by strong anti-*B. burgdorferi* antibodies, which were weak in mice infected with *bb0238∆IM* isolates (Fig 1c). Similarly, unlike the other isolates, we were unable to regrow *bb0238∆IM* isolates from infected murine tissues, as shown by culture analysis (Fig 1d). Low levels of *B. burgdorferi* in a host could be undetectable by molecular or microbiological methods but can be acquired by and detected in tick vectors via a method known as xenodiagnosis [19]. Therefore, parallel groups of WT-, *bb0238∆IM-*, *and bba57-* infected mice were parasitized by naïve *Ixodes scapularis* ticks, and *B. burgdorferi* levels in fed ticks were then measured by RT-qPCR. The results confirmed that, unlike the wild type or control isolates, the PPI-deficient mutants were undetectable in fed ticks (Fig 1e). Overall, these results emphasize that the BB0238-BB0323 PPI is essential to the persistence of *B. burgdorferi* in mice and its acquisition by ticks from murine hosts.

The inability of *bb0238∆IM* isolates to persist in mice and be acquired by feeding ticks is likely because the loss of BB0238-BB0323 interactions interferes with the mutual stability and levels of both proteins. We therefore explored whether such effects on proteins in the PPI-deficient spirochetes could be visualized in a simple whole cellular dot-blot assay. Wild type or *bb0238∆IM* isolates were grown to equal density, and serially diluted lysates prepared from whole cells were blotted onto nitrocellulose and immunoblotted with anti-BB0323 antibody. The PPI-deficient mutants displayed reduced levels of BB0323, with undetectable levels in a lower dilution, suggesting the PPI event could be examined by a cellular dot-blot assay (Fig 1f). To extend our published studies showing that BB0323-BB0238 interactions stabilize both interacting proteins [11,12,15], we measured the stability of BB0238 protein in absence or presence of BB0323 by assessing its melting temperature (T_m_) through differential scanning fluorimetry (DSF), specifically via nanoDSF experiments. We showed that the addition of increasing amount of BB0323 to BB0238 leads to enhancement of the T_m_ values in dose response suggesting more stabilized BB0238 in presence of BB0323 (Fig 1g). Of note, BB0323 does have very low intrinsic fluorescence at the tested concentrations and, therefore, does not influence the fluorescence signal of BB0238 (S1 Fig).

### Crystal structure of BB0238-BB0323 complex reveals unique organization of the PPI interface

**Fig 2:**
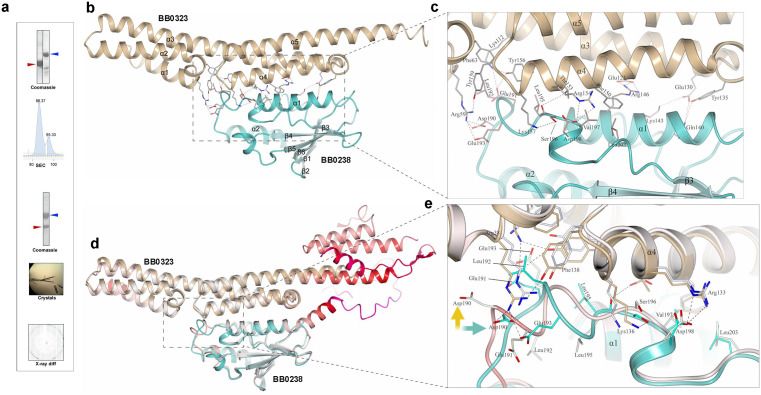
Structure of BB0238-BB0323 complex solved via X-ray crystallography. **(a)** Crystallization of BB0238-BB0323 complex. Recombinant BB0323 and BB0238, indicated by blue and red arrows, respectively, are shown before and after complex formation as performed by size-exclusion chromatography (SEC). Representative crystals of the BB0238-BB0323 complex and a diffraction image are presented. **(b)** Crystal structure of *B. burgdorferi* protein-protein complex between BB0238_118-256_ (cyan) and BB0323_26-210_ (pale brown). BB0238 and BB0323 residues involved in complex formation are indicated as stick models. Both α-helices in BB0238 are marked as α1 and α2, while the six β-strands are noted as β1 to β6. The five α-helices in BB0323 are indicated from α1 to α5. **(c)** A closeup view of the boxed area from panel B, showing the residues involved in the BB0238-BB0323 complex formation. BB0238 is illustrated in cyan and BB0323 in pale brown. **(d)** Superimposed crystal structure of BB0238 (cyan) and BB0323 (pale brown) complex with the AlphaFold 3-predicted structure of BB0238_22-256_-BB0323_22-236_ complex (RMSD: 0.92 Å). In the predicted structure, both protomers are colored according to the local distance difference test (pLDDT), ranging from red (low confidence) to white (high confidence). **(e)** The interface between BB0238 and BB0323 is depicted as a magnified image from panel d, showing the superimposed crystal structure of BB0238_118-256_-BB0323_26-210_ (cyan and pale brown, respectively) with the AlphaFold 3-predicted model, which is colored according to the pLDDT values (red: low confidence; white: high confidence). Conformational divergences of specific residues are observed, leading to the absence of several interactions in the AlphaFold 3-predicted model relative to the crystal structure; one example, Asp190, is shown by cyan and yellow arrows, respectively.

To gain structural insights into the BB0238-BB0323 interaction at the 3D interface, which could be crucial for the future development of therapeutics targeting this essential PPI event, we performed X-ray crystallographic studies of the protein complex. An empirical approach was deemed necessary, as AlphaFold 3 predicted the complex structure with only moderate accuracy (ipTM < 0.7). The interaction site of these multidomain proteins is known to encompass residues 120–130 and 22–200 of BB0238 and BB0323 [14], respectively (Fig 1a). Therefore, for solving the BB0238-BB0323 complex structure, we generated the recombinant BB0238 encompassing the C-terminal domain residues 118–256, and BB0323 covering residues 26–210 (Fig 2a). In the crystal structure of the BB0238_118–256_-BB0323_26–210_ complex, BB0238 was built for residues 132–256, but BB0323 structure was assessed using residues 43–201. The electron density was not observed for the first few N-terminal and the last C-terminal residues, suggesting their flexible nature. The results are consistent with the previously solved crystal structures of individual BB0238 and BB0323 proteins, where these residues were also not observed [11,17]. We show that the PPI covers a relatively large interface area (Fig 2b), which is typical of PPI contacts in protein complexes from other organisms [20]. As detected from PDBePISA quaternary structure analysis [21], the buried PPI surface area spans 2,040 Å^2^ that involves ionic bonds where lysine and arginine interactions with aspartic acid and glutamic acid residues dominate. A closer analysis revealed that the complex formation is also stabilized by multiple hydrophobic interactions; specifically, BB0238 residues Leu195, Val197, and Leu203 form a hydrophobic interaction with residues Phe63 and Phe150 in BB0323 (Fig 2c). In BB0323, the residues involved in complex formation are found in different locations and include the loop region between α1 and α2 (Arg59, Phe63), α3 (Lys112, Glu123, Glu130), the loop region between α3 and α4 (Tyr135), α4 (Phe150, Thr153, Arg154, Tyr156, Lys157), and the loop region between α4 and α5 (Tyr159). In BB0238, most of the residues involved in complex formation are located in the loop region between α2 and β3 (Asp190, Glu191, Leu192, Leu195, Ser196, Asp198, Val197, Asp198, Leu203), and the remaining three residues are found on α1 (Gln140, Lys143, Arg146). Whereas the previously predicted interaction site in BB0238 covering residues 120–130 [15] was not seen in the crystal structure, we produced an additional recombinant protein, BB0238_132–256_, which was also crystallized in the complex with BB0323. The resulting BB0238_132–256_-BB0323_26–210_ complex structure was solved at 3.6 Å resolution and was a high-confidence match with the BB0238_118–256_-BB0323_26–210_ complex (RMSD value of 0.41 Å) (S2 Fig). Therefore, we speculate that mutating residues 120–130 to all alanine residues in a previous study [15] may have altered the mutual positions of the N- and C-terminal domains, thereby disrupting the interaction. The AlphaFold 3 prediction of the BB0238_22–256_-BB0323_22–236_ complex revealed that residues 120–130 in BB0238 form a low-confidence region with an average predicted local distance difference test (pLDDT) value of 52.9 (Fig 2d). Additionally, the remainder of the N-terminal region of BB0238 (residues 22–120) also shows low confidence (average pLDDT value of 52.8), suggesting its inherent flexibility. Notably, residues 186–198, which cover the loop region between α2 and β3 in BB0238 and are involved in complex formation according to the crystal structure, adopt a different conformation and show a decreased confidence score (average pLDDT value of 81.9) in the predicted model. Consequently, several interactions observed in the crystal structure, such as those involving Asp190 and Glu191 in BB0238, are absent in the predicted model due to conformational divergence (Fig 2e).

Electrostatic potentials as calculated by APBS [22] revealed a distinct negatively charged surface covering the approximate location of BB0238, while a positively charged patch covering was observed on the opposite side of the protein-protein complex (S2 Fig). Although the precise mechanism by which the BB0238-BB0323 complex contributes to microbial persistence in mammalian hosts or transmission between ticks and mammals remains unclear, the presence of these distinct charge patches suggests a potential role in the interaction process.

Taken together, the above series of studies shows that the BB0238-BB0323 PPI complex features a ~ 1,000 Å^2^ surface area, stabilized primarily by ionic and hydrophobic interactions, with residues from BB0238 and BB0323 distributed across the loop and helix regions contributing to the interface.

### Quantitative high-throughput screening (qHTS) identifies BB0238-BB0323 PPI inhibitors

**Fig 3:**
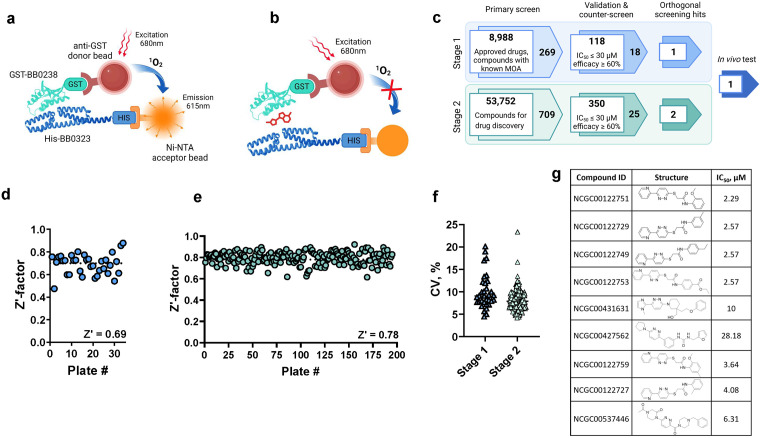
Inhibitors of BB0323-BB0238 interaction are identified through a quantitative high-throughput screening campaign. **(a-b)** Schematic representation [81] of the AlphaLISA PPI assay. The interaction of GST-tagged BB0238 and His-tagged BB0323 proteins conjugated to anti-GST donor beads and nickel chelate acceptor beads, respectively, brings the beads in close proximity, which enables, upon excitation, the energy transfer from the donor beads to the acceptor beads, resulting in a fluorescence signal at 615 nm **(a)**. The disruption of the PPI by small molecules leads to a loss of signal **(b)**. **(c)** Discovery flow chart [81] for small molecule inhibitors of BB0323-BB0238 PPI. The screening campaign was separated into stage 1 and stage 2 based on the features of the screened libraries. The chart shows the key screening phases, the numbers of compounds that were tested or selected at each step, and specific selection criteria for a given phase. **(d-f)** Stage 1 and stage 2 screening statistics: Z’ factors of individual plates in the primary screenings from stage 1 **(d)** and stage 2 **(e)**, and the coefficients of variation (CV) of individual plates **(f)**. **(g)** Compound structures of Cluster 7 (for details see text) along with their BB0323-BB0238 PPI inhibitory activity as evaluated in the primary assay.

We next explored if the disruption of this complex can serve as a novel therapeutic strategy against Lyme disease. To discover small molecule inhibitors of the BB0238-BB0323 PPI complex via qHTS, we utilized an amplified luminescent proximity homogeneous assay linked immunosorbent assay (AlphaLISA) technology with anti-GST donor and anti-His acceptor Alpha beads in combination with recombinantly produced GST-tagged BB0238 (GST-BB0238) and His-tagged BB0323 (His-BB0323) proteins (Fig 3a). If the BB0238-BB0323 PPI is disrupted by a small molecule, the distance between the beads will not be close enough to allow singlet oxygen transfer from the donor beads to the acceptor beads, resulting in Alpha signal decrease (Fig 3b). To ensure that the high-affinity interaction between BB0238 and BB0323 remains intact when the proteins are tagged (S3 Fig), we measured the binding of GST-BB0238 to His-BB0323 by microscale thermophoresis (MST). The PPI was confirmed with an equilibrium dissociation constant (K_D_) of 2 ± 0.17 µM (S3 Fig).

Next, we developed the PPI assay in 384-well plates and verified the signal specificity using different proteins (S3 Fig), as well as competition experiments with untagged BB0238 protein (S3 Fig). To enable automated high-throughput screening, the PPI assay was miniaturized and optimized to a 1536-well plate format. Based on cross-titration experiments of BB0323 and BB0238 proteins, 15 nM His-BB0323 and 15 nM GST-BB0238 in combination with 5 µg/mL of each bead was determined to be the optimal condition for the assay (S3 Fig).

Subsequently, we executed two stages of qHTS using the optimized AlphaLISA BB0238-BB0323 PPI assay (Fig 3c). In stage 1, a total of 8,988 compounds from libraries of approved drugs, investigational drugs with known mechanisms of action, and natural products were screened using a four-point dilution series with final compound concentrations ranging from 18.3 nM to 91.7 μM. During stage 2, we screened 53,752 compounds from in-house libraries designed for drug discovery. The BB0238-BB0323 PPI assay showed high performance and robustness, as indicated by the high Z’ factor scores of the primary screen in both stage 1 (Fig 3d) and stage 2 (Fig 3e) (0.69 and 0.78, respectively), as well as the low coefficients of variation (CV) with averages of 9.9% and 8.1%, respectively (Fig 3f). After applying filters to eliminate redox cyclers and promiscuous compounds [23], 269 compounds from stage 1 and 709 compounds from stage 2 in curve class 1–3 [24] were selected for follow-up experiments based on their potency (half maximum inhibitory concentration [IC_50_] at or below 30 µM) and efficacy (≥ 60% PPI inhibition). These compounds were retested in the primary AlphaLISA PPI assay in 11-point 1:3 dilution series, with final compound concentrations ranging from 0.89 nM to 52.9 μM, to confirm their inhibitory activity. 118 compounds (43.87%) from stage 1 and 350 compounds (49.37%) from stage 2 were validated to be active with IC_50_ ≤ 30 µM and efficacy ≥ 60% (Fig 3c).

Since the stage 2 screening libraries contain chemotypes with shared structural features, we performed clustering on the 350 active compounds using StarDrop, applying a 70% similarity threshold based on common substructures. This analysis identified 47 clusters, each containing at least two compounds, along with 196 singletons, highlighting significant structural diversity (S1 Table). Among these, 13 clusters comprised four or more compounds (S4 Fig), emphasizing recurring structural motifs. Notably, Cluster 14, consisting of 20 compounds, exhibited an IC_50_ range of 0.062 to 16.24 µM, suggesting a tractable structure-activity relationship (SAR). Likewise, Cluster 7, containing 9 compounds, (S1 Table and S4 Fig) displayed an IC_50_ range of 2.95 to 4.57 µM, further indicating a clear SAR within the cluster. Fig 3g displays the structures and inhibitory activities of Cluster 7 compounds in comparison to some structurally similar singletons. These findings suggest that certain structural motifs within the clusters contribute positively or negatively to bioactivity profiles. Additionally, the presence of both active and inactive analogs further confirms the activity associated with these hits.

The primary hit compounds were also tested in a counter screen assay to detect false positive hits which can interfere with the Alpha technology by acting as singlet oxygen quenchers, color quenchers, or light scatterers, or by disrupting the binding of AlphaLISA beads to the affinity tags. We used His-tagged GST protein as a control in an identical assay format to the primary screening instead of His-BB0323 and GST-BB0238. In parallel, these compounds were tested for their cytotoxicity against human embryonic kidney (HEK293) cells.

Based on the results of the validation, counter screen, and cytotoxicity experiments, we cherry-picked compounds for orthogonal experiments in *B. burgdorferi* to assess compounds that are effective in reducing the BB0238-BB0323 PPI within live bacteria. Due to the low throughput of the cellular assay, we focused on molecules with drug-like features and representative compounds from the promising clusters identified above. A total of 43 compounds – 18 from stage 1 and 25 from stage 2 (Fig 3c and S2 Table) – were chosen for the cell-based experiments detailed below.

### Select compounds inhibit BB0238-BB0323 PPI within *B. burgdorferi*

**Fig 4:**
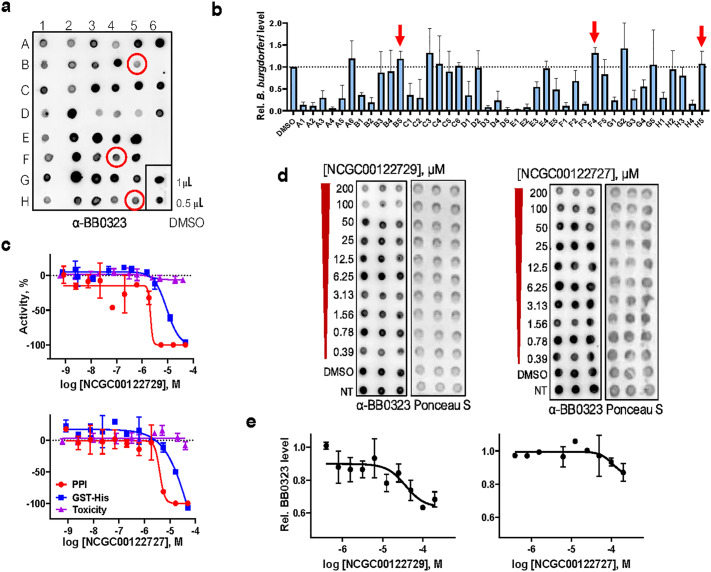
Performance of hit molecules blocking BB0323-BB0238 PPI in primary and cell-based orthogonal assays. **(a-b)** Assessment of hit molecules in bacterial cell-based orthogonal assays at a single dose (200 µM). A representative dot-blot image (n = 3) for the determination of relative BB0323 levels in compound-treated cells using anti-BB0323 antibody **(a)** and results of the regrowth assay (mean ± SD of n = 3) **(b)**. Three hit molecules that show PPI inhibition, as indicated by reduced BB0323 levels, but with minimal impact on spirochete growth are highlighted by red circles and corresponding arrows. **(c-e)** Performance of compounds NCGC00122729 and NCGC00122727 in hit evaluation assays. Dose response curves are shown for hit molecules in the BB0323-BB0238 PPI primary assay, His-GST counter screen, and cytotoxicity assay **(c)**. Both compounds were further tested using dot-blot assays in dose response to determine their inhibitory activity in spirochetes. Representative dot-blot and Ponceau S staining images **(d)**. Densitometric analysis of the dot-blots normalized to the total protein determined by Ponceau staining were plotted against the concentration to determine the IC_50_. Data values are shown as mean ± SD, n = 3 **(e)**.

We previously showed that the loss of the BB0238-BB0323 PPI impacts the posttranslational stability of both proteins, as well as the spirochetes’ ability to persist in mice, although it does not affect microbial growth in culture [12]. Therefore, we developed a strategy to screen for compounds that can enter live borrelial cells in culture and inhibit the native interaction of BB0323 and BB0238, as assessed by measuring cellular levels of BB0323 in a dot-blot assay, without impacting microbial viability, as examined by a re-growth assay of *B. burgdorferi* (S5 Fig). Due to the lower throughput of the cellular assays, the cherry-picked compounds were tested initially at a single concentration of 200 µM. Fig 4a shows a representative image of the dot-blot experiment, where the relative level of BB0323 was observed after borrelial cells were treated with compounds. In parallel, we performed a regrowth assay [25], where aliquots of the treated spirochetes were cultured in fresh media and the cells were counted after 96 h incubation (Fig 4b). We identified three compounds which substantially reduced the level of endogenous BB0323 but did not affect *B. burgdorferi* growth (marked in Fig 4a and 4b). Interestingly, two of these active compounds – NCGC00122729 and NCGC00122727 – were from Cluster 7 and showed structural similarities and PPI inhibitory activities in the biochemical assay discussed above (Fig 3g). The performances of these two compounds in the inhibition of PPI, counter screen, and mammalian cytotoxicity assays are shown in Fig 4c, reflecting a modest toxicity against cells of mammalian origin.

Next, we tested various doses of the hit compounds in the BB0323 dot-blot assay where we show NCGC00122729 and NCGC00122727 in live *B. burgdorferi* exhibited an IC_50_ of 37.2 µM and 109 µM, respectively (Fig 4d, 4e). The third active compound in *B. burgdorferi,* lomibuvir, was selected further for *in vivo* studies in mice based on the data from all the assays, as further detailed below.

### Lomibuvir, as a proof-of-concept hit, partially impacts *B. burgdorferi* infection in a tick-transmitted murine model of Lyme disease

**Fig 5:**
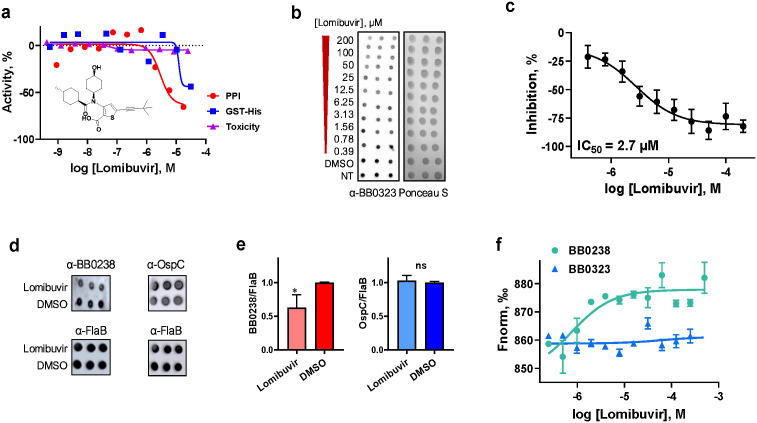
Lomibuvir inhibits BB0323-BB0238 PPI in biochemical and spirochete-based assays. The compound’s structure and dose-response curves from the BB0323-BB0238 PPI inhibitory assay (IC_50_ = 3.11 µM), counter screen, and cytotoxicity assays are presented. Data values are shown as mean ± SD, n = 3 **(a)**. Lomibuvir activity was tested in spirochetes via BB0323 dot-blot assay in dose-response. Ponceau staining of the blot was performed for total protein determination **(b)**. PPI inhibition was determined by the densitometric analysis of BB0323 levels from the dot-blot shown in (b) normalized to total protein amount **(c)**. **(d-e)** Specificity of lomibuvir’s inhibitory effect on BB0323-BB0238 interaction. The upper panels show dot-blots developed against anti-BB0238 or anti-OspC, while the lower panels were probed with FlaB antibodies as loading controls **(d)**. Graphical presentation is shown of the densitometric analysis of blots from independent experiments (n = 3) **(e)**. *p < 0.05. **(f)** Mechanism of action of lomibuvir. The compound specifically binds to BB0238 with a K_D_ of 761.3 ± 4.2 nM, but does not bind to BB0323, as measured by MST.

Lomibuvir, a hit molecule from the stage 1 screen and an investigational drug against hepatitis C virus, displayed robust activity against the BB0238-BB0323 PPI, with an IC_50_ value of 3.11 µM in the primary assay, and no toxicity in human cells (Fig 5a). Notably, lomibuvir exhibited the most potent activity in reducing the native BB0323 levels in *B. burgdorferi* among the tested compounds. The dose-response analysis of lomibuvir activity in the cellular dot-blot assay revealed an IC_50_ value of 2.7 µM (Fig 5b, 5c). As the disruption of the BB0238-BB0323 PPI, such as by targeted mutation [15], impairs the posttranslational stability of both proteins, we next assessed whether the expected PPI inhibition via lomibuvir also impacts BB0238 levels, together with its reduction of BB0323 levels as shown in Fig 5b. Dot-blot assays using antigen-specific antibodies showed that the treatment of spirochete cells with lomibuvir also impaired levels of BB0238, but not control borrelial proteins, such as OspC (Fig 5d, 5e). Further, we performed MST assays to evaluate target engagement and confirmed that lomibuvir binds specifically to BB0238 with high affinity (K_D_ of 761.3 ± 4.2 nM), but not to BB0323 (Fig 5f).

**Fig 6:**
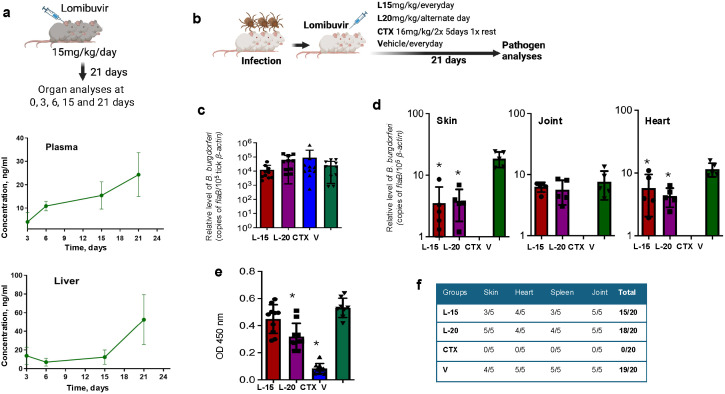
Lomibuvir impairs *B. burgdorferi* infection in a tick-transmitted murine model of Lyme borreliosis. **(a)** Pharmacokinetics study showing lomibuvir bioavailability and release profile in murine tissues. The top image [81] shows the experimental strategy, while the middle and bottom panels show the pharmacokinetic effects on plasma and liver samples, respectively. The data indicate the concentration of the compound at each timepoint. **(b-f)** *In vivo* testing of lomibuvir efficacy in treating murine *B. burgdorferi* infection. A schematic model [81] depicts the assessment of lomibuvir treatment using the tick-borne murine model for *B. burgdorferi* transmission **(b)**. Nymphal *I. scapularis* ticks were microinjected with spirochetes and allowed to parasitize mice; after feeding to repletion, their *B. burgdorferi* levels were assessed by RT-qPCR **(c)**. One day after the conclusion of treatment, *B. burgdorferi* levels in murine skin, joint, and heart samples were assessed by RT-qPCR **(d)**, while murine sera were used to analyze serological responses in mice via ELISA **(e)**; *p < 0.05. Culture analysis of infected mouse tissues after treatment is depicted **(f)**. Various collected tissues were incubated in BSK media for up to 4 weeks and the presence of viable spirochetes was detected using dark field microscopy. The results are representative of two independent animal experiments.

Based on the orthogonal assay data for the three hit compounds, as described above, lomibuvir was selected for further proof-of-concept *in vivo* studies in mice. First, we examined the pharmacokinetic properties of lomibuvir in the murine model by measuring the compound concentration at various post-injection times and target sites. A total of 18 C3H mice were administered 15 mg/kg of lomibuvir consecutively for 21 days, which were monitored closely for signs of toxicity, as observed by deviations from normal growth and social behavior of the animals. Following compound treatment, three mice per timepoint were euthanized at 0, 3, 6, 15, and 21 days (Fig 6a). Blood and organ samples, such as skin, liver, heart, bladder, and joint tissues, were collected and processed for the detection of lomibuvir via LC-MS/MS. The compound was detectable in all organs, and the concentration was increased at later stages of treatment, such as between 15–21 days, as shown in the blood (plasma) and liver samples (Fig 6a).

As lomibuvir was tolerated in mice at the dose tested above, we next assessed its efficacy against *B. burgdorferi* infection using a tick-borne murine model of Lyme disease. As depicted in Fig 6b, four separate groups of C3H mice were challenged with *B. burgdorferi-*infected ticks and then intraperitoneally treated with 15 mg/kg of lomibuvir daily, 20 mg/kg of lomibuvir on every other day as an alternate dose regimen, ceftriaxone (positive control), or vehicle (negative control), over the course of 21 days. One day following the final treatment, spirochete burdens in repleted ticks and in various murine tissues were evaluated by RT-qPCR analysis. The results demonstrated that *B. burgdorferi* levels in repleted ticks were comparable in each group (Fig 6c), confirming that mice across all groups were exposed to similar levels of pathogens. Analysis of murine samples showed a significant reduction of pathogen levels in the skin and heart tissues of both compound-treated groups (both doses), as compared to vehicle controls, whereas spirochete burdens in the joint tissues did not differ significantly (Fig 6d). Serological analysis, via ELISA with *B. burgdorferi* lysates against antisera from infected mice, suggested that infection was significantly lower in the 20 mg/kg lomibuvir-treated group, but not in the 15 mg/kg group (Fig 6e). Despite lower pathogen burdens in multiple organs and lower antibody responses in compound-treated mice, culture analyses showed that *B. burgdorferi* was recovered from a majority of the tested murine tissues after treatment with lomibuvir (Fig 6f). Overall, these findings indicate that lomibuvir, in the doses studied, could lower *B. burgdorferi* burdens in mice in a tissue-specific manner.

## Discussion

*B. burgdorferi* is uniquely adapted to survive in a complex enzootic cycle involving ticks and mammals, and can establish persistent infections in susceptible vertebrate hosts, including humans [7]. Unlike conventional bacteria, these spirochetes reflect an unusual biology, such as atypical cellular architecture, lack of many classical recognition molecules, and a segmented genome that encodes many proteins of unknown functions [8–10]. These properties likely contribute to the ability of *B. burgdorferi* to survive in disparate tissue environments in arthropod vectors and vertebrate hosts, establishing persistent infections that can render standard antibiotic treatments less effective. In fact, *B. burgdorferi* are known to persist in many animals following antibiotic treatment in experimental settings [26], although there is limited evidence of such persistence in humans [19,27]. Nevertheless, due to the absence of human Lyme vaccines, and the fact that a subset of patients treated with standard-of-care antibiotics often continue to experience relapsing symptoms, there is an unmet and urgent need for innovative therapeutic approaches. A recent study explored hygromycin A as a selective antibiotic against *B. burgdorferi* [28], but its utility in treating human Lyme disease requires further validation. Notably, its mode of action [29] is the inhibition of conserved bacterial function and protein synthesis, which is similar to that of doxycycline, the most common antibiotic currently prescribed for Lyme disease [30], raising questions about the potential advantages of hygromycin A over existing treatments. More recently, *B. burgdorferi* has specifically shown to be sensitive to piperacillin [31], although the mechanism of this antibiotic is similar to other beta-lactams. Thus, the limitations of current antibiotic therapies further highlight the complexity of addressing *B. burgdorferi* infections and calls for the discovery of antimicrobials with novel modes of action.

A relatively new class of antimicrobials [32], also known as anti-virulence compounds or pathoblockers [33], that interact with non-essential bacterial targets has emerged as an attractive therapeutic option. These compounds are represented by inhibitors of virulence-conferring factors or pathways, such as quorum sensing [34], biofilm [35], secretion systems [36], tissue penetration [37], or intracellular survival [38]. In *B. burgdorferi,* a series of unique genes that encode membrane proteins of unknown functions are shown to be non-essential for spirochete growth in culture, yet play critical roles in microbial virulence *in vivo*, promoting persistence in the host [12,13,16,18,39–42]. We have shown that while the BB0238-BB0323 interaction is non-essential for microbial growth and survival *in vitro*, it is indispensable to spirochete infectivity [12,13,15,16], thus suggesting a novel therapeutic strategy to utilize inhibitors that target essential PPIs, or ones with other unique modes of action. While the discovery of small molecule PPI inhibitors has been historically considered difficult, advancements in recent years have led to successful examples [43,44], including ones involving bacteria [45]. As BB0323 and BB0238 are highly conserved within the borrelial clade yet display no homology to other proteins, inhibitors of their PPI should have minimal off-target effects. Although mutational studies highlighted the crucial roles of both proteins in spirochete biology [16,46], infectivity [12,13,47] and immune evasion [11], given their lack of homology to known proteins in the sequence databases, the complete structures and exact biochemical functions of BB0323 and BB0238 remain unknown and require empirical studies. For example, the experimental structure of the BB0238-BB0323 complex, which we have reported herein, revealed that the loop orientation differs significantly from the AlphaFold 3-predicted model, including interactions between residues with biological relevance. Knowledge of the crystal structures of these proteins and their interacting complex will be crucial for drug discovery efforts through structure-based drug design and for revealing the mechanism of action of potential hit compounds. Analogous structural studies to guide PPI-based drug development are well documented, such as the development of pilicide derivatives that inhibit bacterial pilus assembly by competing with the usher-chaperone interaction [48], as well as the development of a Type 2 diabetes drug, piragliatin, which is a glucose kinase activator that binds in an allosteric site [49]. To facilitate future medicinal chemistry efforts, we solved the structural details of the BB0238-BB0323 complex and identified potential PPI inhibitor compounds. Initial characterizations led to the shortlisting of one hit with known antiviral activity, lomibuvir [50]. Lomibuvir serves as an example of a new class of molecules that affect spirochete infection *in vivo* by acting as PPI inhibitor. Although lomibuvir demonstrated only partial success in clearing *B. burgdorferi* infection in the host and is considerably inferior to ceftriaxone treatment, additional studies exploring various doses, routes, and extended timepoints, as well as its optimization via medicinal chemistry, could be explored to enhance its efficacy while maintaining an acceptable safety profile. In addition, the detailed characterization of other BB0238-BB0323 PPI inhibitors, such as NCGC00122729 and NCGC00122727 that were identified in our screens, could also contribute to the discovery of additional antimicrobials against Lyme disease. These compounds share a common core and present structure-activity relationship. Taken together, these molecules as well as uncharacterized hits, along with our optimized small molecule screening platform and resources, could be expected to contribute to the development of future novel drug discovery efforts.

The biological activities of many proteins are exerted through discrete PPIs, which play essential roles in biology [51]. While complex eukaryotic proteomes can form an estimated 505,000 PPIs [52,53], comparisons of assembled cross-species bacterial interactomes identified at least 52,000 unique PPIs across 349 different bacterial species and strains [54], with approximately 30% of these interactions involving proteins of unknown functions and thus having obscure biological significances. Some of the bacterial PPIs have been characterized where corresponding small molecule inhibitors are also identified [54,55]. However, compared to the few dozens of small molecule PPI modulators that have been identified for eukaryotes, which predominantly target cancer, immune responses, autoimmune disorders, or viruses, with several being in Phase I, II, and III clinical trials [20,56], such progress against bacterial PPI targets is lagging relatively behind. Nevertheless, as bacterial PPIs are often involved in essential cellular processes, small molecule inhibitors are being identified that modulate relevant activities such as division and replication, transcription, membrane biogenesis, toxin–antitoxin systems, and lipolysis [57]. These recent developments highlight the concept that the targeting of PPIs represents an attractive therapeutic strategy, as they may offer a new and unconventional point of intervention that could inform the development of novel anti-infective drugs against bacterial pathogens [45,57,58].

Lomibuvir (previously known as VX-222 or VCH-222) is a potent allosteric inhibitor of the RNA-dependent RNA polymerase (NS5B) [50], where the compound binds to the thumb domain (site II) of the polymerase in Hepatitis C virus with an IC_50_ in the sub-micromolar range [59]. Unlike eukaryotes, which possess at least three forms of RNA polymerases, bacteria rely on a single RNA polymerase (RNAP) that is responsible for transcription. This bacterial enzyme has previously been explored as a target for the discovery of inhibitors targeting PPIs [60–62]. Whether lomibuvir can also bind or modulate spirochete RNA polymerase activity, or precisely how it binds BB0238 and inhibits an essential PPI in *B. burgdorferi*, warrants further investigation. However, as we have identified a subset of the amino acid residues in the BB0238-BB0323 interface that confer the majority of the binding energy towards PPI formation, it is possible to identify additional structurally diverse small molecule compounds that can block the BB0238-BB0323 PPI by binding to alternate residues of the proteins, by further investigation of additional hits and compounds identified in our current screen. Our study therefore serves as a critical first step towards new antimicrobial discovery by identifying these hotspots that would allow the targeting of potential “druggable” sites, paving the way for the rational design of new therapeutic strategies against *B. burgdorferi* infection.

Lomibuvir was previously shown to demonstrate clinical efficacy [50,63] and was subjected to Phase 1b clinical trials approximately a decade ago, where the compound treatment resulted in a reduction of viral loads, although its development was later discontinued due to resistant variants and competition with other drug regimens [64]. Preliminary metabolism and pharmacokinetics data are available for *in vitro* lomibuvir treatment, using cultured primary hepatocytes from humans or nonclinical species like rats, dogs, and monkeys [65]; however, its PK/PD (pharmacokinetic-pharmacodynamic) properties are undisclosed, including in mice. Our current study agrees with earlier preliminary data on VX-222/VCH-222 showing its oral bioavailability in rats and the accumulation of compound in the liver [65]. Lomibuvir displays favorable ADME properties in terms of permeability and metabolic pathways and lacks evidence for the potential of reactive intermediates [65], although it remains to be investigated whether the further optimization of a treatment regimen, as well as the exploration of refined chemical scaffolds or its combinatorial use with mainstream [30] or other conventional antibiotics [66], would provide added benefits. Furthermore, future efforts are warranted to investigate the additional compounds identified in our study that inhibit the BB0238-BB0323 PPI, along with other essential PPI events identified in *B. burgdorferi* involving additional proteins [67], including BB0108 that interacts with BB0238 [11], as these could represent viable opportunities to explore novel antimicrobial therapeutic targets. This approach of targeting an essential microbial PPI holds promise as a novel treatment strategy against Lyme disease, for which more effective and comprehensive antibiotic therapies are needed.

## Materials and methods

### Ethics statement

The University of Maryland College Park Institutional Animal Care and Use Committee (IACUC), as well as the University of Maryland College Park Institutional Biosafety Committee (IBC), reviewed and approved the experiments involving animals and biohazards, respectively.

### Bacteria, mice, and ticks

The *B. burgdorferi* B31 isolates A3 [16] and 297 [15], grown in Barbour–Stoenner–Kelly H (BSK) medium, were used in the present study. The generation of *B. burgdorferi* mutants in our laboratory, including isolates deficient in the BB0238 interaction motif (*bb0238*∆IM) [15] or *bba57*- mutants [18], were described previously. Hatched egg masses originating from individual female *I. scapularis* ticks were purchased from Oklahoma State University Tick Rearing Facility. The larval ticks were fed on mice, after which they were collected and allowed to molt to nymphs. Four- to six-week-old female C3H/HeN mice were bred in-house or purchased from Charles River Laboratories.

### Nucleic acid isolation and RT-qPCR

Isolation of RNA, reverse transcription, and PCR were performed using published procedures [40]. Briefly, total RNA was isolated from ticks and murine tissues using TRIzol Reagent (Invitrogen) and treated with DNase I (NEB). The cDNA was transcribed from RNA using the SuperScript VILO cDNA Synthesis Kit (ThermoFisher Scientific) and analyzed by qPCR using Bio-Rad thermocycler with SYBR Select Master Mix (ThermoFisher Scientific). *B. burgdorferi flaB* cDNA copies were measured and normalized to tick or mouse *β-actin* levels to quantify spirochetes, as detailed [40].

### Recombinant protein expression, purification, and antibody production

Protein induction and purification, as well as the production of polyclonal antibodies, were carried out using previously described methods [12,15]. Recombinant proteins GST-BB0238, His-BB0238, and His-BB0323 were expressed in *E. coli* BL21 (DE3) with either pET28a, pGEX-6P-1, or pET303 expression vectors. Proteins were dialyzed to remove impurities and concentrated via Amicon Ultra Centrifugal Filter units (Millipore). For antibody production, mice were injected subcutaneously three times with 10 µg of recombinant proteins in Freund’s adjuvant, as detailed previously [68]. BB0238_118–256_, BB0238_132–256_, and BB0323_26–210_ production for crystallization experiments was carried out as previously described for both proteins [11,17]. Briefly, the amplified genes were cloned into the pETm-11 expression vector and expressed in *E. coli* BL21 (DE3) as recombinant proteins containing a 6xHis tag. Proteins were purified using Ni-NTA agarose resin (Qiagen) on a gravity-flow column before mixing BB0238 and BB0323 at a molar ratio of 1:2. The mixture was then loaded onto a pre-equilibrated HiLoad 16/600 Superdex 200 prep-grade column (GE Healthcare, USA). The peak fraction containing the BB0238-BB0323 complex was concentrated using an Amicon centrifugal filter unit (Millipore).

### Crystallization, data collection, and structure determination

The crystallization trials for the BB0238_118–256_-BB0323_26–210_ and BB0238_132–256_-BB0323_26–210_ complexes were set up by a Tecan Freedom EVO100 workstation (Tecan Group Ltd.) in 96-well sitting-drop plates by combining 0.4 μL of protein with 0.4 μL of precipitant solution from JCSG-plus and structure screen 1&2 sparse matrix screens (Molecular Dimensions Ltd., UK). The crystals used for diffraction data collection for the BB0238_118–256_-BB0323_26–210_ complex were grown in a precipitant solution containing 24% PEG 1500 and 20% glycerol. For the BB0238_132–256_-BB0323_26–210_ complex, crystals were obtained in a solution containing 0.01 M NiCl2, 0.1 M Tris (pH 8.5), and 20% PEG 2000 MME. Before data collection, the BB0238_132–256_-BB0323_26–210_ crystals were harvested using 20% glycerol as a cryoprotectant. X-ray diffraction data were collected at the MX beamline instrument BL 14.1 at Helmholtz-Zentrum, Berlin [69]. Reflections were indexed using XDS and scaled with AIMLESS from the CCP4 suite [70]. The complex structure was determined by molecular replacement using Phaser [71], with the crystal structures of BB0238 (PDB ID 8P33) and BB0323 (PDB ID 6RJX) as search models. Protein models were built using BUCCANEER [72], followed by manual rebuilding in COOT [73]. Crystallographic refinement was performed using REFMAC5 [74]. A summary of the data collection, refinement, and validation statistics for the BB0238_118–256_-BB0323_26–210_ and BB0238_132–256_-BB0323_26–210_ complexes is provided in S3 Table.

### Immunoblotting

The western blotting analyses were performed as described [16,67]. For the dot-blot assay, one µL of the borrelial cell lysate was dotted on semi-dried nitrocellulose membrane (0.45 µm pore size) that was pre-wetted with phosphate-buffered saline (PBS; pH 7.5). The dots were allowed to dry for 10 minutes and then the membrane was blocked with 3% milk in PBS pH 7.5 with 0.05% Tween-20 (PBST), followed by overnight incubation with antibody against BB0323 N-terminal protein (1:3000), as generated previously [12,13], followed by HRP conjugated anti-mouse antibody (1:10,000). Blots were developed using enhanced chemiluminescence HRP substrate (Thermo Fisher Inc.) and the densities of the blots were further analyzed by ImageJ 2.1.0 software.

The total protein load on the membrane was evaluated by Ponceau S staining (Thermo Fisher Inc.) following manufacturers protocol. To calculate the IC_50_ values in the dose-response experiments, the dot-blot densities and the corresponding Ponceau S-stained dots were analyzed by ImageJ. The dot-blot densities were normalized to the total protein amount and the data from the DMSO-treated sample set as 1. The resulting data were fitted to a sigmoidal dose response curve using four-parameter Hill equation.

### Enzyme-linked immunosorbent assay (ELISA)

ELISA was performed as detailed [75] with minor modifications. Briefly, 96-well plates (Nunc) were coated with 500 ng of spirochete lysates overnight at 4°C and blocked with 1% BSA in PBS for 1 hour at room temperature (RT). The wells were incubated with serum from *Borrelia*-infected mice at 1:3000 concentration and incubated for 1 hour at 37°C, followed by the addition of HRP-conjugated goat anti-mouse IgG (1:10,000) and incubation for 1 hour at RT. The signal was developed with TMB peroxidase substrate reagents, stopped with 0.1 N hydrochloric acid, and read at 450 nm. The assay was done in triplicates at least two times.

### Differential scanning fluorescence (nanoDSF)

Thermal unfolding studies were performed using the NanoTemper Prometheus NT.48 with PR.ThermControl software, using our published method [11]. Briefly, 20 µM BB0238 and varying concentrations of BB0323 proteins individually or in combination were resuspended in assay buffer and incubated for 30 minutes at RT before loading into NanoTemper standard capillaries. Excitation energy was set so that at least one protein gave 2500 counts in the 330 and 350 nm channels, and thermal ramp from 25 to 90 °C at 4 °C/minute ramp speed. The Tm, or midpoint of the thermal unfolding sigmoid, was determined by taking the global minimum or maximum of the first derivative of each protein’s thermal unfolding curve, while initial folding values were derived from the ratio of 350nm/330nm before the thermal ramp was applied.

### Microscale thermophoresis (MST)

MST assay was performed using a Monolith NT.Automated instrument (NanoTemper Technologies), as detailed earlier [11]. The His-tagged proteins were labeled with NanoTemper RED-tris-NTA 2^nd^ generation dye. For assessment of the interaction between His-BB0323 and GST-BB0238, 10 nM of labeled His-BB0323 was incubated with varying concentrations of GST-BB0238 for 30 min at RT. For small molecule binding experiments, fluorescently labeled proteins at concentrations of 10 nM were mixed with compounds at 12-point 1:1 dilution series. After incubation, the mixture was loaded into standard capillaries (NanoTemper Technologies) and analyzed at 10% excitation power and medium MST power. MO.Affinity Analysis software was used to analyze the data and calculate K_D_ values.

### Quantitative high-throughput screen (qHTS) for BB00323-BB0238 PPI assay

All screening operations were performed on a fully integrated robotic system (Kalypsys Inc., San Diego, CA). The assay protocol is summarized in Table 1. The reagents were diluted in AlphaLISA assay buffer (PBS pH 7.4, 0.05% Tween-20). Briefly, 2.5 μL His-BB0323 (final concentration of 15 nM) was dispensed into a 1536-well solid white plate (Greiner Bio-One) using BioRaptr liquid handling equipment, and 23 nL of compounds, or DMSO as control, were then pinned or acoustically dispensed. After a 15-minute incubation, 1.5 µL of GST-BB0238 (final concentration of 15 nM) was dispensed, and plates were incubated for 1 hour. Then, 500 nL of nickel chelate AlphaLISA acceptor beads (5 μg/mL final concentration; Perkin Elmer Inc.) were added. After 30 min incubation, 500 nL glutathione donor beads (5 μg/mL final concentration; Perkin Elmer Inc.) were added, followed by another 30 min incubation. All incubation steps were performed at RT, and the plates were centrifuged for 15 sec at 1000 x *g* after each reagent dispensing step. The AlphaLISA signal was detected using a PheraStar plate reader. Column 2, containing both proteins and DMSO, served as a no-inhibitor control. Columns 3 and 4 contained single protein alone and were used as no-PPI controls. The signal from each plate was normalized against no-inhibitor control (set as 0%) and no-PPI controls (set as 100%), and the resulting data showing the percentages of inhibition were fitted to a sigmoidal dose response curve using four-parameter Hill equation.

**Table 1:** BB0323-BB0238 PPI assay protocol.

Step	Parameter	Value	Description
1a 1b	Buffer or BB0323	2.5 µL	**Columns 1:** PBS, pH 7.4 + 0.05% Tween20 **Columns 2–48:** 15 nM His-BB0323 (final concentration)
2a 2b	Control or Compounds	23 nL	**Columns 1–4** DMSO **Columns 5–48** Library compounds
3	Time	15 min	Incubation at room temperature
4a 4b	Buffer or BB0238	1.5 µL	**Column 1:** PBS, pH 7.4 + 0.05% Tween20 **Columns 2 and 5–48:** 15 nM GST-BB0238 (final concentration)
5	Time	1 h	Incubation at room temperature
6	Acceptor	0.5 µL	**Columns 1–48:** 5 µg/mL nickel chelate Beads (final concentration)
7	Time	30 min	Incubation at room temperature
8	Donor	0.5 µL	**Columns 1–48:** 5 µg/mL Glutathione beads (final concentration)
9	Time	30 min	Incubation at room temperature in the dark
12	Detector	AlphaLISA	PheraStar plate reader (module AlphaLISA 680/615, exc. 50ms)

### His-GST counter screen assay

The counter screen assay was performed with recombinant His-GST protein (Sigma Aldrich) following the AlphaLISA PPI protocol described above and as shown in Table 1. Instead of dispensing His-BB0323 and GST-BB0238, the same amounts of His-GST and AlphaLISA assay buffer, respectively, were dispensed into the plates.

### Mammalian cytotoxicity assay

Cytotoxicity assays were conducted with CellTiter-Glo reagent (Promega) according to the manufacturer’s protocol. Briefly, 1x10^3^ HEK293 cells were dispensed into 1536-well solid white plates (6 μL per well) and treated with 25 nL of compound, bortezomib positive cell-killing control, or DMSO vehicle controls for 24 hours at 37°C. Then, 4 μL of CellTiter-Glo reagent was added to the wells and the plates were incubated at RT for 10 min. Luminescence was detected using a PerkinElmer ViewLux microplate reader equipped with clear filters (10 sec exposure, 2X binning), and data was normalized using bortezomib and DMSO control signal.

### *B. burgdorferi* cellular assay

The schematics of the cell-based orthogonal assay are shown in S5 Fig. *B. burgdorferi* cultures were grown to the mid-log phase in BSK medium at 33°C. 100 µL of 1x10^6^ cells/well were seeded in each well of 96-well microtiter plates. Compounds at a single concentration or in serial dilutions in DMSO were added to the duplicate wells, and the plates were incubated at 37°C for 16–18 hours. For control, the same volume of DMSO (vehicle control) or media (no treatment control) were used in parallel wells. After incubation, one µL of culture was added to fresh 200 µL of BSK medium to observe the re-growth of *B. burgdorferi* after 96 hours. The rest of the culture was pelleted, and the cells were lysed with 20 µL PBS containing 1% Triton X-100 and vortexing for 30 minutes on ice. A dot-blot assay was performed with one µL of the cell lysate, using procedures as detailed above. Anti-BB0323 antibody was used as the method of protein detection. The assay was performed three times with at least two technical replicates each.

### Formulation of compound for in vivo studies

For the formulation of lomibuvir, 5 mg compound in a clear vial was mixed with 50 µL of N-Methyl-2-pyrrolidone (NMP), sonicated and vortexed to obtain a clear solution. 550 µL polyethylene glycol 300 (PEG300) was added to the mixture and sonicated to mix well. Finally, 400 µL of water was added and mixed well using sonication to obtain a clear solution.

### Pharmacokinetics study

Six separate groups of animals (three C3H/HeN mice per group) were intraperitoneally injected with 15 mg/kg of lomibuvir daily for 21 days. Mice were euthanized at specific timepoints between 0–21 days, and blood and organ samples, including skin, liver, heart, bladder, and joint tissues, were collected and stored at -80°C until further analysis. Samples were analyzed via LC-MS/MS using a commercial source (at ChemoGenics BioPharma, NC). For analysis, sterile PBS was added to the tissues, four times their weight, and subjected to homogenization with an Omni Bead Ruptor instrument. 50 µL of tissue samples were kept in 96-well plates, where 150 µL of acetonitrile was added. The plate was sonicated for 30 seconds and centrifuged. The supernatant was collected on a clean plate. Five µL of the samples were injected into the LC-MS/MS system at 50°C. Data analysis via Analyst software (Version 1.7.1) was used to capture the data and for further analysis.

### *In vivo* studies of the biological significance of BB0323-BB0238 interaction

To assess the impact of the PPI on the persistence of *B. burgdorferi* in murine hosts, groups of C3H/HeN mice (three animals per group) were injected intradermally with an equal number (10^5^ cells per mouse) of wild type (WT), BB0323-BB0238 interaction-deficient mutants (*bb0238∆IM*), or *bba57* deletion mutants as control (*bba57*-), as detailed [18]. Two weeks after injection, serum was collected from the mice and antibody responses were assessed against wild type *B. burgdorferi* lysates to confirm the establishment of infection. Nine weeks after injection, the mice were infested with naïve ticks (seven nymphs/mouse). Ticks were collected after complete repletion and processed [16] for RT-qPCR analysis to compare the levels of acquired spirochetes. Murine tissues were assessed by serological analysis also cultured in BSK media to assess the presence of viable spirochetes, as detailed [13,76].

### Proof-of-concept efficacy studies

To evaluate the efficacy of lomibuvir against *B. burgdorferi* infection using the tick-borne mouse model of Lyme borreliosis, cohorts of naïve nymphal ticks were used that had been equally infected with wild-type *B. burgdorferi* via microinjection, as detailed [77]. Four separate groups of C3H mice (five animals per group) were allowed to be engorged by infected ticks (seven nymphs per mouse). After tick repletion, the animals were intraperitoneally treated over a period of 21 days with compound or controls: 15 mg/kg of lomibuvir per day, 20 mg/kg of lomibuvir on alternate days, 16 mg/kg of ceftriaxone per day (as positive control [78]), or vehicle (as negative control). After the conclusion of treatment, *B. burgdorferi* levels in fed ticks were determined by qPCR, while pathogen burdens in mouse tissues were assessed via ELISA, qPCR, and culture analyses, as detailed [79,80].

### Statistical analysis

All the results are presented as means ± standard errors of the mean (SEM). GraphPad Prism software, version 4.0-9.0, was used to measure statistically significant differences, as evaluated using Student’s t-test or ANOVA.

## Supporting information

S1 Fig Analyses of BB0238 and BB0323 by nanoDSF. nanoDSF spectra at 350 nm **(a)** and 330 nm **(b)** of individual BB0238 and BB0323 proteins or mixed together along their respective first derivatives (lower panel).
(PDF)

S2 Fig Structure of BB0323-BB0238. **(a)** Complex of BB0323-BB0238. Superimposed crystal structures of BB0238_132–256_-BB0323_26–210_ complex (PDB ID 9Q9V; gold) with BB0238_118–256_- BB0323_26–210_ complex (PDB ID 9QAA; orange; RMSD value of 0.41 Å). Both α-helices in BB0238 are marked as α1 and α2, while the six β-strands as β1 to β6. The five α-helices in BB0323 are indicated from α1 to α5. **(b)** Electrostatic surface potentials of *B. burgdorferi* BB0238-BB0323 complex. The protein-protein complex structure is illustrated from two different angles rotated by 180°. The surface contour levels were set to – 1 kT/e (red) and + 1 kT/e (blue).
(PDF)

S3 Fig Interaction of BB0238 and BB0323. **(a)** Purity and identity validation of the recombinant GST-tagged BB0238 and His-tagged BB0323 proteins by SDS-PAGE and Coomassie staining. **(b)** The binding of GST-BB0238 to His-BB0323 was measured by MST and resulted in an equilibrium dissociation constant (K_D_) of 2 ± 0.17 µM. **(c-d)** Specificity of Alpha signal induced by BB0238 and BB0323 interaction. Alpha signal is only detectable with His-BB0323 and its interacting partner GST-BB0238, but not with buffer or the tested GST-tagged control proteins (domain of unknown function [DUF] and HtrA) **(c)**. Alpha signal between His-BB0323 and GST-BB0238 is affected by the presence of untagged BB0238 **(d)**. **(e)** Assay miniaturization into 1536-well plate format. Optimal assay conditions were determined by cross-titration of BB0323 and BB0238.
(PDF)

S4 Fig Stage 2 hit molecules. Structural clusters with four or more compounds identified from the validated stage 2 hit molecules.
(PDF)

S5 Fig Schematics of the cell-based orthogonal assay. The image [81] shows the strategy to identify cell-permeable inhibitors using an anti-BB0323 dot-blot assay and a *Borrelia* regrowth assay.
(PDF)

S1 Table List of active compounds from Stage 2 screening used for structural clustering with their IDs, structures, SMILES, corresponding cluster numbers, numbers of compounds per cluster and their activity and efficacy in the AlphaLISA PPI assay. (XLSX)

S2 Table Active compounds from Stage 1 and Stage 2 picked for the cell-based assays based on their activity and efficacy in the primary PPI assay, counter-screen and cytotoxicity in HEK293 cells with their ID, sample name and molecular structure. (XLSX)

S3 Table Statistics for data and structure quality. The statistical information for the indicated protein complexes is shown.
(PDF)
