# Supplementary figures and images for: Targeting of interaction between BB0323-BB0238 informs new paradigms in Lyme disease therapeutics
Source: PLoS Pathog. 2026 Jan 2;22(1):e1013805. doi: 10.1371/journal.ppat.1013805 (PMC12758692; doi:10.1371/journal.ppat.1013805)

S1 Fig

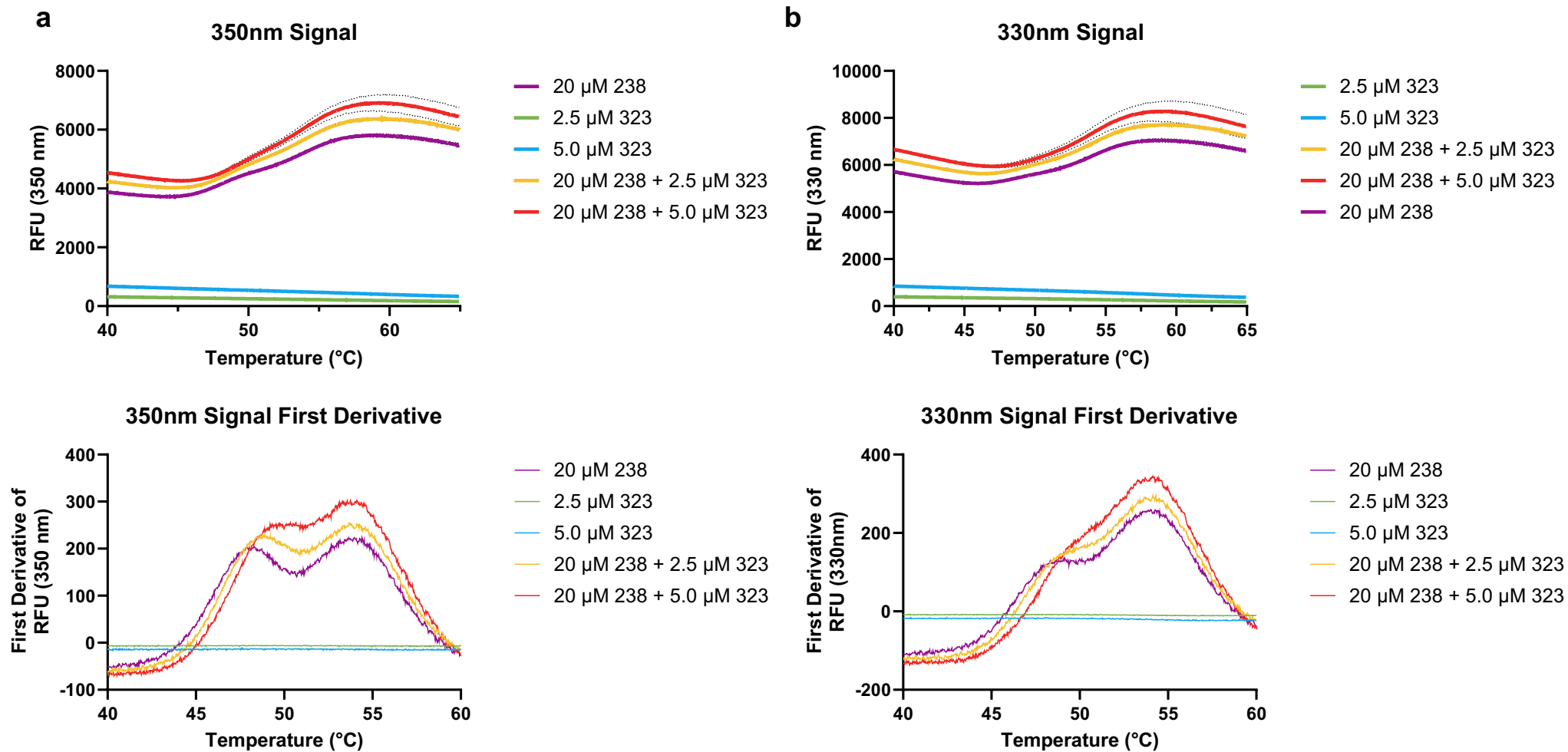

Supplement: S1 Fig — nanoDSF spectra at 350 nm (a) and 330 nm (b) of individual BB0238 and BB0323 proteins or mixed together along their respective first derivatives (lower panel). (PDF) [file ppat.1013805.s001.pdf]

S2 Fig

**a**

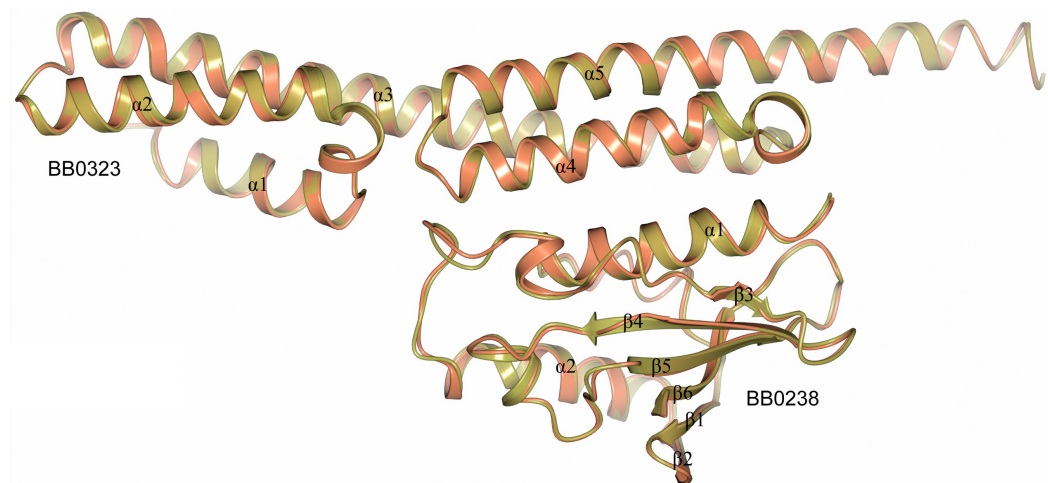

BB0238<sub>118-256</sub>-BB0323<sub>26-210</sub> complex: 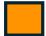  
BB0238<sub>132-256</sub>-BB0323<sub>26-210</sub> complex: 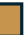

**b**

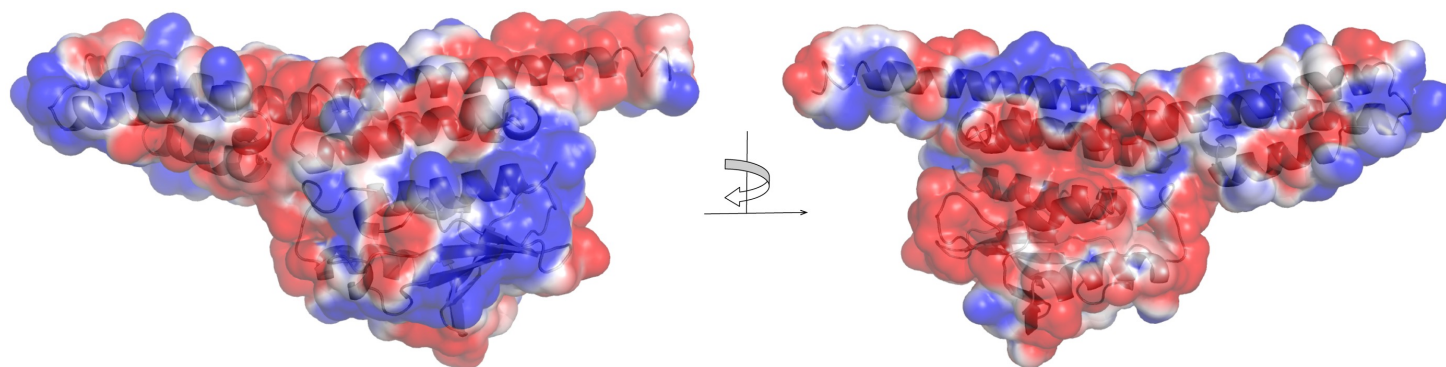

Supplement: S2 Fig — (a) Complex of BB0323-BB0238. Superimposed crystal structures of BB0238132–256-BB032326–210 complex (PDB ID 9Q9V; gold) with BB0238118–256- BB032326–210 complex (PDB ID 9QAA; orange; RMSD value of 0.41 Å). Both α-helices in BB0238 are marked as α1 and α2, while the six β-strands as β1 to β6. The five α-helices in BB0323 are indicated from α1 to α5. (b) Electrostatic surface potentials of B. burgdorferi BB0238-BB0323 complex. The protein-protein complex structure is illustrated from two different angles rotated by 180°. The surface contour levels were set to – 1 kT/e (red) and + 1 kT/e (blue). (PDF) [file ppat.1013805.s002.pdf]

S3 Fig

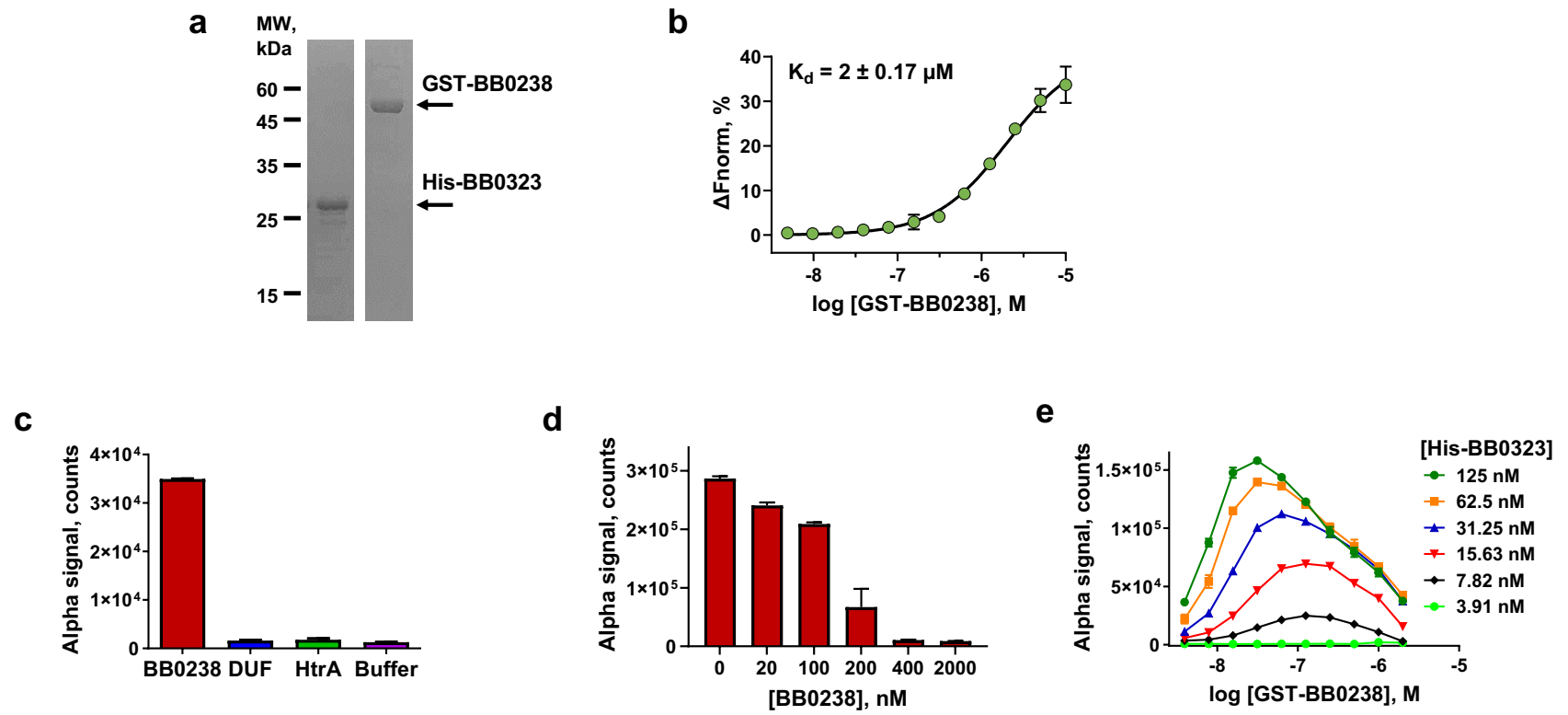

Supplement: S3 Fig — (a) Purity and identity validation of the recombinant GST-tagged BB0238 and His-tagged BB0323 proteins by SDS-PAGE and Coomassie staining. (b) The binding of GST-BB0238 to His-BB0323 was measured by MST and resulted in an equilibrium dissociation constant (KD) of 2 ± 0.17 µM. (c-d) Specificity of Alpha signal induced by BB0238 and BB0323 interaction. Alpha signal is only detectable with His-BB0323 and its interacting partner GST-BB0238, but not with buffer or the tested GST-tagged control proteins (domain of unknown function [DUF] and HtrA) (c). Alpha signal between His-BB0323 and GST-BB0238 is affected by the presence of untagged BB0238 (d). (e) Assay miniaturization into 1536-well plate format. Optimal assay conditions were determined by cross-titration of BB0323 and BB0238. (PDF) [file ppat.1013805.s003.pdf]

S4 Fig

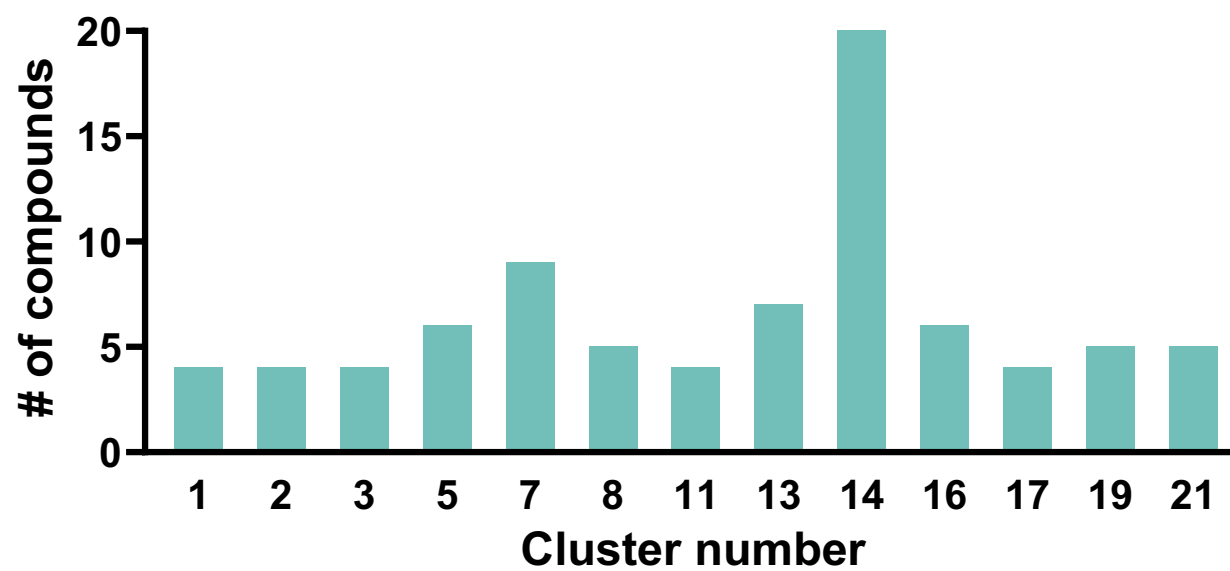

Supplement: S4 Fig — Structural clusters with four or more compounds identified from the validated stage 2 hit molecules. (PDF) [file ppat.1013805.s004.pdf]

S5 Fig

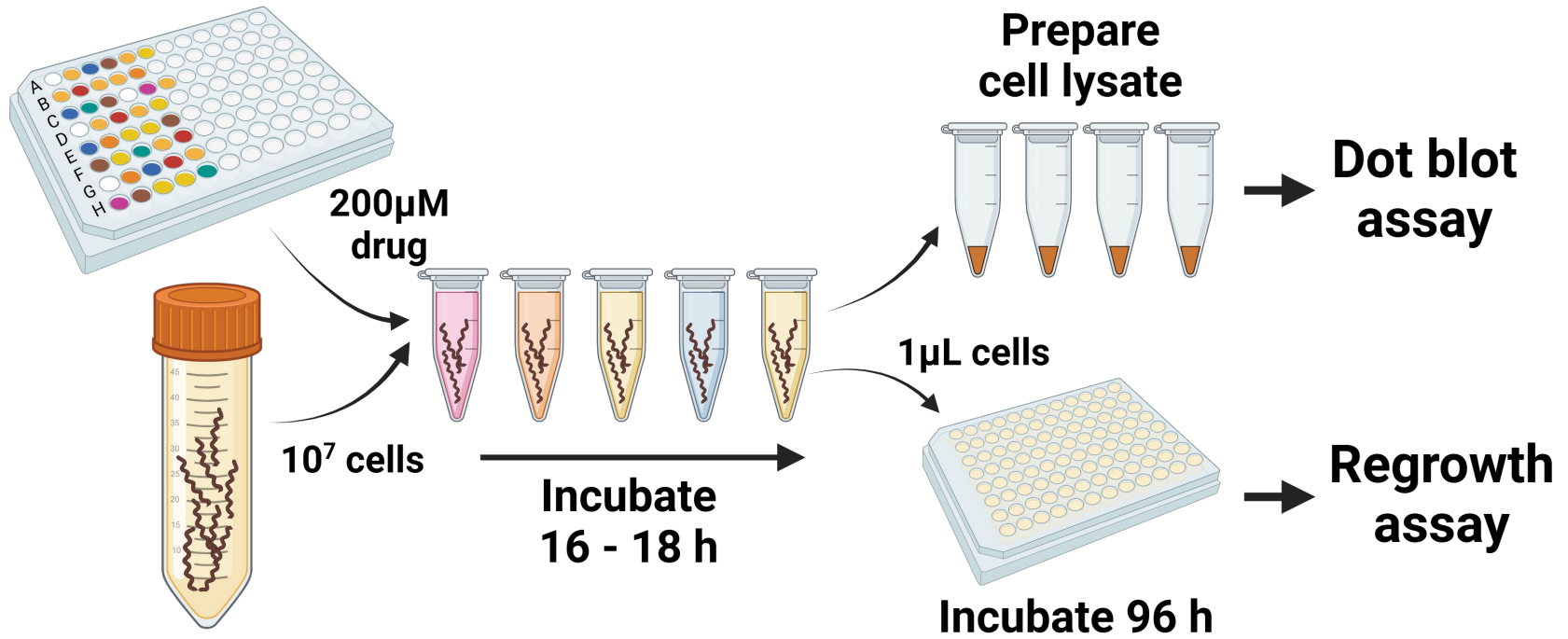

Supplement: S5 Fig — The image [81] shows the strategy to identify cell-permeable inhibitors using an anti-BB0323 dot-blot assay and a Borrelia regrowth assay. (PDF) [file ppat.1013805.s005.pdf]
