# Supplementary material for: Targeting of interaction between BB0323-BB0238 informs new paradigms in Lyme disease therapeutics
Source: PLoS Pathog. 2026 Jan 2;22(1):e1013805. doi: 10.1371/journal.ppat.1013805 (PMC12758692; doi:10.1371/journal.ppat.1013805)
Supplement: S3 Table — The statistical information for the indicated protein complexes is shown. (PDF) [file ppat.1013805.s008.pdf]

S3 Table. Statistics for Data and Structure Quality.

Dataset	$BB0238_{118\text{-}256}$-$BB0323_{26\text{-}210}$	$BB0238_{132\text{-}256}$-$BB0323_{26\text{-}210}$
**PDB ID**	9QAA	9Q9V
**Space group**	P65	P61
**Unit cell dimensions**		
**a (Å)**	129.74	130.76
**b (Å)**	129.74	130.76
**c (Å)**	41.24	38.94
**Wavelength (Å)**	0.9762	0.9184
**Resolution (Å)**	64.87-3.50	42.80-3.60
**Highest resolution bin (Å)**	3.83-3.50	3.94-3.60
**No. of reflections**	56951	22584
**No. of unique reflections**	5206	1084
**Completeness (%)**	100.0 (100.0)	99.9 (100.0)
R_{merge}	0.07 (0.38)	0.10 (0.54)
$I/\sigma(I)$	16.3 (6.6)	18.9 (6.9)
**Multiplicity**	10.9 (11.3)	19.9 (20.8)
**Refinement**		
R_{work}	0.233 (0.279)	0.199 (0.249)
R_{free}	0.297 (0.411)	0.335 (0.375)
**Average B-factor (Å^2)**		
**Overall**	103.9	118.4
**From Wilson plot**	104.0	118.5
**No. of atoms**		
**Protein**	2313	2237
**Water**	0	0
**RMS deviations from ideal**		
**Bond lengths (Å)**	0.005	0.005
**Bond angles (°)**	1.455	1.445
**Ramachandran outliers (%)**		
**Residues in most favored regions (%)**	81.34	83.33
**Residues in allowed regions (%)**	18.29	16.67
**Outliers (%)**	0.37	0.00

Values in parentheses are for the highest resolution bin.
